# Molecular alterations induced by a high-fat high-fiber diet in porcine adipose tissues: variations according to the anatomical fat location

**DOI:** 10.1186/s12864-016-2438-3

**Published:** 2016-02-18

**Authors:** Florence Gondret, Annie Vincent, Magalie Houée-Bigot, Anne Siegel, Sandrine Lagarrigue, Isabelle Louveau, David Causeur

**Affiliations:** INRA, UMR1348 Pegase, F-35590 Saint-Gilles, France; Agrocampus-Ouest, UMR1348 Pegase, F-35000 Rennes, France; Agrocampus-Ouest, UMR6625 IRMAR, F-35000 Rennes, France; CNRS-Université de Rennes 1-INRIA, UMR6074 IRISA, Campus de Beaulieu, 35042 Rennes, Cedex France

**Keywords:** Adipose tissue, Dietary fiber, High-fat diet, Gene module, Multiple factor analysis, Transcriptome, Upstream regulators

## Abstract

**Background:**

Changing the energy and nutrient source for growing animals may be an effective way of limiting adipose tissue expansion, a response which may depend on the genetic background of the animals. This study aims to describe the transcriptional modulations present in the adipose tissues of two pig lines divergently selected for residual feed intake which were either fed a high-fat high-fiber (HF) diet or an isocaloric low-fat high-starch diet (LF).

**Results:**

Transcriptomic analysis using a porcine microarray was performed on 48 pigs (*n* = *12* per diet and per line) in both perirenal (PRAT) and subcutaneous (SCAT) adipose tissues. There was no interaction between diet and line on either adiposity or transcriptional profiles, so that the diet effect was inferred independently of the line. Irrespective of line, the relative weights of the two fat depots were lower in HF pigs than in LF pigs after 58 days on dietary treatment. In the two adipose tissues, the most apparent effect of the HF diet was the down-regulation of several genes associated with the ubiquitin-proteasome system, which therefore may be associated with dietary-induced modulations in genes acting in apoptotic and cell cycle regulatory pathways. Genes involved in glucose metabolic processes were also down-regulated by the HF diet, with no significant variation or decreased expression of important lipid-related genes such as the low-density lipoprotein receptor and leptin in the two fat pads. The master regulators of glucose and fatty acid homeostasis *SREBF1* and *MLXIPL*, and peroxisome proliferator-activated receptor (*PPAR)δ* and its heterodimeric partner *RXRA* were down-regulated by the HF diet. *PPARγ* which has pleiotropic functions including lipid metabolism and adipocyte differentiation, was however up-regulated by this diet in PRAT and SCAT. Dietary-related modulations in the expression of genes associated with immunity and inflammation were mainly revealed in PRAT.

**Conclusion:**

A high-fat high-fiber diet depressed glucose and lipid anabolic molecular pathways, thus counteracting adipose tissue expansion. Interaction effects between dietary intake of fiber and lipids on gene expression may modulate innate immunity and inflammation, a response which is of interest with regard to chronic inflammation and its adverse effects on health and performance.

**Electronic supplementary material:**

The online version of this article (doi:10.1186/s12864-016-2438-3) contains supplementary material, which is available to authorized users.

## Background

White adipose tissue (AT) has a central role in various physiological processes related to lipid metabolism and energy homeostasis in mammals [1, 2] and is considered to be a significant contributor to systemic low-grade inflammation [3]. Adipose tissue development is triggered by the imbalance between energy intake and expenditure and most related studies have exploited high-fat hyper-caloric diets to generate adipose tissue expansion and document molecular alterations [4–6]. The nutrient source is also important in determining adiposity. Noticeably, dietary fibers, which are poorly digested by monogastric animals, are associated with energy dilution of the diet, hence reducing the adiposity that accompanies the chronic intake of high levels of fat [7, 8]. This reduction results from a lower food intake, enhanced insulin sensitivity, increased energy expenditure, and changes in expression levels of some genes involved in lipid anabolism and fatty acid β-oxidation, in different organs [7, 9]. However, the diversity in molecular pathways in AT which can be altered by a high-fat high-fiber diet remains still to be clarified, together with the ways by which these changes relate to adiposity variations. The pig is recognized as an experimental model to investigate genetic and mechanistic aspects of human disease such as obesity [10–12], and is a leading source of animal protein in human diets. The introduction of more fiber to cereal-based diets formulated for pigs has regained interest due to both economic considerations and the potential benefits on health and welfare [13]. While, the addition of fat to a fiber-rich diet is also required to retain a dietary energy value compatible with good animal performance [14]. Therefore, the primary objective of this study was to decipher the transcriptomic changes that take place in AT when pigs received either a high-fat high-fiber diet or an isocaloric low-fat high-starch diet, thus changing energy source and nutrients rather than energy content in diet. The availability of pig lines divergently selected for residual feed intake (RFI), a measure of feed efficiency, offers an additional opportunity to consider line-associated differences in basal metabolism and energy expenditure [15, 16] when reasoning body composition and dietary responses. Both independent and possible interaction effects between diet and line on the AT transcriptional profiles have then to be considered.

The anatomical location of AT is also important when analyzing the molecular responses to diet. In many species [17, 18] including pigs [19, 20], differences in adipogenic factors, responses to hormones, metabolic properties or secretion of inflammatory cytokines have been observed between subcutaneous AT and visceral ATs which are located within the abdominal cavity. Whereas subcutaneous fat grows at the same rate as total body fat, perirenal (visceral) fat grows faster in pigs; thus, the distinction between these two anatomical fat sites is also economically important. Hence, similarities and particularities in the molecular responses to diet of subcutaneous and perivisceral ATs are worthy of investigation. It has recently been shown that AT expansion was reduced at the subcutaneous and perirenal locations when growing pigs of two divergent RFI lines were fed a high-fat high-fiber diet as compared with pigs fed an isocaloric low-fat high-starch diet [21]. Molecular mechanisms and processes associated with both diet and line in the dorsal subcutaneous and perirenal ATs are explored in the present study. Pathways common to both ATs and also specificities within each AT in the responses to diet, are highlighted using dedicated integrated statistical methods.

## Results

### Summary of phenotypic data

Forty-eight growing pigs of two lines divergently selected on RFI, a measure of feed efficiency, were fed diets formulated at isocaloric and isoproteic bases but differing in energy source and nutrients (lipids and fibers *vs.* starch). Full description of performance of experimental pigs (*n = 12* per line and per diet) after 58 days of dietary treatment can be found in an associated paper [21] and are briefly summarized here. Importantly, there was no interaction between diet and line on performance and body composition. Irrespective of RFI line, pigs fed the high-fat high-fiber (HF) diet ate 12 % less (*p* < 0.001) than pigs fed the low-fat low-fiber starch-based (LF) diet during the trial and they developed less body fat, as illustrated by 1.4-fold lower proportions of ATs at the perirenal (%PRAT) and dorsal subcutaneous (%SCAT) locations. The differences between diets were significant when the adiposity data was corrected for individual daily feed intake, indicating that AT development was triggered not only by differences in feed intake between pigs but also by the nutrient composition. Irrespective of diet, divergent selection for RFI did not affect AT weights in this experiment (Table 1).

**Table 1:** Growth and adiposity as affected by diet and line

Diet	HF		LF		Statistics	
Line	Low	High	Low	High	*p*-diet	*p*-line
Pigs^a^, n	12	12	12	12		
Initial age (d)	73	73	75	75	0.77	0.04
Initial weight (kg)	25.7	26.6	26.5	27.1	0.62	0.54
Final age (d)	132	131	134	133	0.49	0.12
Final body weight (kg)	72.8	69.0	82.7	77.6	<0.001	0.008
Feed intake (kg/d)	2.13	2.12	2.45	2.40	<0.001	0.45
Gain to food ratio	0.38	0.34	0.39	0.36	0.13	<0.001
Adipose tissue mass (% body weight)						
Perirenal AT	0.59	0.54	0.83	0.83	<0.001	0.46
Subcutaneous AT	3.90	4.10	5.90	5.70	<0.001	0.41

^a^Pigs were fed a high-fat high-fiber diet (HF) or a low-fat high-starch diet (LF). Duration of the feeding trial was 58.5 ± 0.5 days for all pigs. Two divergent pig lines that have been selected over eight generations for residual feed intake (RFI), a measure of feed efficiency, were used. Pigs selected for low RFI were the most efficient in the conversion of food to weight gain. There was no significant interaction (*p* > 0.10) between diet and line on performance and adiposity traits. At the end of the trial, weights of perirenal and dorsal subcutaneous adipose tissues (AT) were used as surrogates of body adiposity

### Diet markedly affected adipose tissue molecular profiles

**Fig 1:**
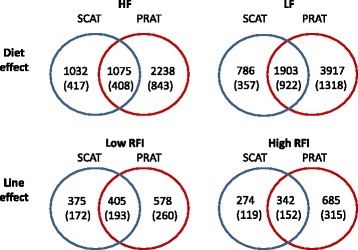
Number of differentially-expressed probes and corresponding unique genes in adipose tissues as affected by diet and line. Microarray data obtained in subcutaneous (SCAT) or in perirenal (PRAT) adipose tissues were separately analyzed for the main effects of diet (HF: high-fat high-fiber vs. LF: low-fat high-starch), line (low RFI: residual feed intake below the average; high RFI: residual feed intake above the average), and the interaction between diet and line. Molecular probes were declared as differentially-expressed between diets or between lines according to cutoffs for fold-change between conditions > |1.1| and *p* < 0.01 (Benjamin-Hochberg adjusted *p*-value < 0.08). Venn diagrams illustrate the number of differentially-expressed probes in each experimental group for SCAT and for PRAT. The corresponding number of differentially-expressed unique genes is indicated into brackets

Microarray analyses were separately performed in PRAT and in SCAT from the 48 experimental pigs. After normalization, the signal intensity was found to be above background noise for 38,142 spots in PRAT and 38,987 spots in SCAT, respectively. The linear model to analyze the log2-transformed signals revealed no significant interaction (p > 0.01) between diet and line on the expression levels of the molecular probes in the two ATs, so that diet and line effects could be inferred independently. The global effects of diet and of line on the number of differentially-expressed probes (DEP) were more pronounced in PRAT than in SCAT (Fig. 1). In the two ATs, diet had also a more pronounced effect on the transcriptome than did the line. Thus, only data related to dietary effects are further considered in this manuscript. By using cut-offs of fold-change between diets > |1.1| and *p*-value < 0.01, while ensuring Benjamini-Hochberg (BH) multiplicity correction of the *p*-values for a control of the FDR at level < 0.08, a total of 3,313 DEP in PRAT and of 2,107 DEP in SCAT were found as over-expressed by HF diet. These corresponded to 1,251 unique differentially-genes (DEG) up-regulated in PRAT and to 825 unique DEG up-regulated in SCAT. Conversely, 4,820 DEP corresponding to 2,440 unique DEG in PRAT, and 2,689 DEP corresponding to 1,279 unique DEG in SCAT were under-expressed by HF diet. Altogether, 1,330 DEG were commonly affected by diet across the two ATs (Fig. 1), which corresponded to 36 % of the DEG in PRAT and 63 % of the DEG in SCAT, respectively.

### Similarities in the molecular responses to diet across two adipose tissues

**Fig 2:**
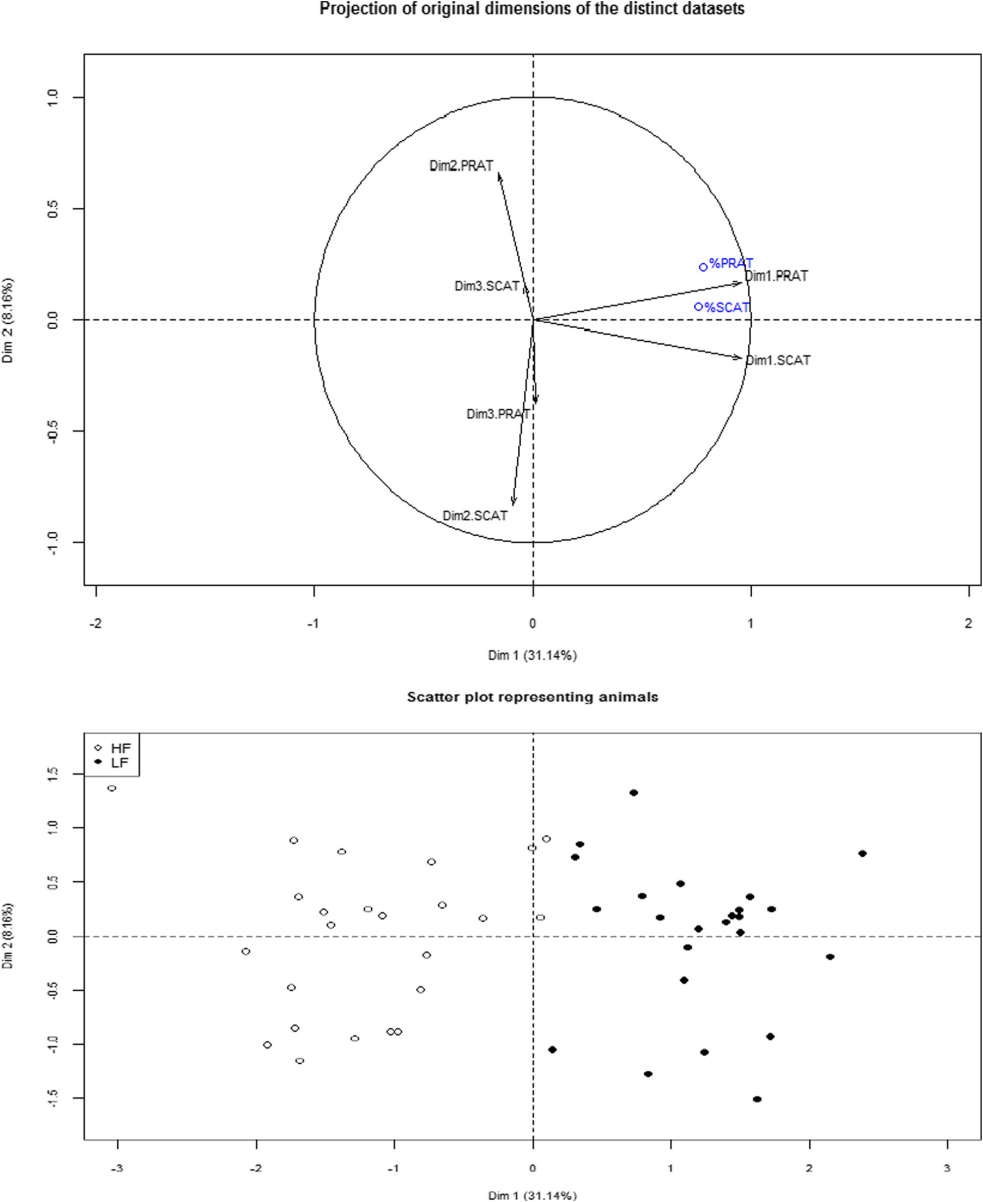
Multi-way datasets analysis: consensus in microarray data relative to dietary effect across two adipose tissues. The first two synthetic variables obtained for the perirenal (Dim_1_PRAT) and subcutaneous adipose tissue (Dim_1_SCAT) transcriptomes were projected in the correlation circle of the multiple factor analysis (MFA), an integrated statistical method used to reveal communalities across separate datasets. Large similarities across adipose tissues can be deduced from molecular variables contributing to the first dimension (Dim1) of MFA (Fig. 1a). Relative weights of perirenal fat (%PRAT) and subcutaneous fat (%SCAT) were superimposed on the plot, showing strong correlation between the molecular probes contributing to Dim1 and adiposity variations. Pigs were represented on the scatter MFA plot (Fig. 1b) and colored following the diet they received (HF: high-fat high-fiber; LF: low-fat high-starch). This shows a well-defined partition of pigs between diets along Dim1

Multiple Factor Analysis (MFA) was performed to derive a common framework of molecular responses to diet and identify the communalities across AT. The basic interest of MFA as a data integration procedure [22] was to ensure that each tissue influence was equally weighed in a manageable number of comprised factors, which can then be related to external phenotypic characteristics. Figure 2 shows the diagnostic MFA plot. The first dimension (Dim1) of the MFA accounted for 31 % of the variation, and correlated significantly to variations in AT weights (r = 0.78 with %PRAT, and r = 0.76 with %SCAT, *p* < 0.001), so that pigs fed the HF diet and pigs fed the LF diet were oppositely represented along Dim1. The second dimension of the MFA (Dim2) explained 8.2 % of the variation and did not clearly separate the data according to diet or adiposity phenotypic traits.

As illustrated (Fig. 2), the two first comprised variables synthesizing the molecular information of the originally-distinct data tables (dim_1_PRAT and dim_1_SCAT, respectively) were projected together along Dim1 in the MFA plot. This means that large similarities between ATs could be inferred by focusing on the DEP that contributed mainly to Dim1. A total of 1,128 DEP showing the highest correlation with Dim1 (r > |0.70|; *p* < 0.001) were thus commonly regulated by diet across the two ATs. The identity of these DEP together with their fold-changes between HF and LF diets are listed in Additional file 1: Table S1 for PRAT and for SCAT, respectively; as indicated in this additional file, all these DEP were altered by diet (BH adjusted *p*-value < 0.05 in PRAT and BH adjusted *p*-value < 0.08 in SCAT). They corresponded to 436 unique differentially-expressed genes (DEG). This statistical approach was thus useful in reducing the number of DEG commonly regulated by diet across the two ATs to those having the strongest correlation with variations in AT weights.

A functional analysis was performed to understand the biological meaning behind this subset of 436 DEG, by using automatic functional annotation tools to identify biological gene ontology (GO) terms and clustering redundant annotation terms in enriched biological pathways. Enrichment (E) score ≥ 1.3 and *p*-value < 0.5 were used to select the top-enriched clusters among these DEG. As indicated in Table 2, the modification-dependent protein catabolic process, intracellular protein transport, coenzyme metabolic process, cellular response to stress, phosphorus metabolic process and hexose metabolic process were the top-enriched biological pathways in the common molecular responses of ATs to diet. The detailed list of genes included in these clusters can be found in Additional file 2: Table S2. To have details on fold-change of these genes between diets, one may also consult Additional file 1: Table S1.

**Table 2:** Enriched biological processes commonly regulated by diet across adipose tissues

Functional annotation of responses to diet	E score^a^	Nb DEG	*p*-value	HF *vs.* LF diet
GO:0019941 ~ modification-dependent protein catabolic process	4.31	34	<0.001	↓: 31 ↑: 3
(GO:0006511 ~ ubiquitin-dependent protein catabolic process)		(21)		
GO:0046907 ~ intracellular transport	3.45	37	<0.001	↓: 29 ↑: 8
(GO:0006886 ~ intracellular protein transport)		(26)		
GO:0006511 ~ ubiquitin-dependent protein catabolic process	2.76	21	<0.001	↓: 20 ↑: 1
(GO:0010498 ~ proteasomal protein catabolic process)		(11)		
GO:0051186 ~ coenzyme metabolic process	1.72	11	0.003	↓: 10 ↑: 1
GO:0033554 ~ cellular response to stress	1.58	28	<0.001	↓: 18 ↑: 10
GO:0016310 ~ phosphorylation	1.42	31	0.017	↓: 21 ↑: 10
GO:0019318 ~ hexose metabolic process	1.32	13	0.003	↓: 13
(GO:0006006 ~ glucose metabolic process)		(11)		
(hsa00620:Pyruvate metabolism)		(7)		
(hsa00010:Glycolysis / Gluconeogenesis)		(5)		
GO:0017038 ~ protein import	1.25	9	0.017	↓: 8 ↑: 1
GO:0009725 ~ response to hormone stimulus	0.99	16	0.042	↓: 10 ↑: 6
GO:0010941 ~ regulation of cell death	0.94	33	0.007	↓: 26 ↑: 7
GO:0000278 ~ mitotic cell cycle	0.66	18	0.010	↓: 15 ↑: 3

^a^The most important genes in the common dietary-related responses across perirenal and subcutaneous adipose tissues and correlating with adiposity variations were considered to list enriched functional clusters. For each cluster based on gene ontology (GO) terms for biological processes and Genomes (KEGG) biological pathways, the enrichment (E) score, the number (Nb) of differentially-expressed genes (DEG) and associated *p*-value were provided. The direction of changes in gene expression levels by the HF diet was symbolized (↑: up-regulated; ↓: down-regulated) together with the number of unique genes concerned by each change

Among protein catabolic process, the ubiquitin-dependent proteolysis was the most represented pathway with a total of 20 genes encoding ubiquitin-conjugating enzymes, different ubiquitin ligases, various ubiquitin-like proteins and different proteasome complex subunits responsible for the degradation of polyubiquitinated proteins, which were down-regulated by the HF diet (Table 2). Similarly, the glucose metabolic process assembled 13 genes, all down-regulated by the HF diet, and coding for enzymes acting in the first step of glucose use (*HK2*), catalyzing glycogen synthesis (*GSK3B, GYS2, UGP2*) or linking glycolysis and tricarboxylic acid cycle (*PDK1, PDHA1, PDHA2, DLAT*). Different factors in coenzyme metabolic processes such as those related to the synthesis of cytosolic acetyl-CoA (*ACLY*) and lipid anabolic process (*ME1, HMGCR*) were also all down-regulated by the HF diet, with the noticeable exception of glutathione S-transferase kappa 1 (*GSTK1*) which functions in cellular detoxification. Conversely, cellular responses to stress included some genes up-regulated in the HF diet (e.g.; *IFI16* and *JMY*, two genes known to interact with p53 in regulating cell cycle progression) as well as genes that were down-regulated by the same diet (e.g.; *CDKN1A*, another regulator of cell cycle progression). Other gene groups with a lower enrichment score (E < 1.3) but *p* < 0.05 could be also regarded as potentially interesting in deciphering the molecular mechanisms modulated by the diet, i.e. the response to hormone stimulus, regulation of cell death and cell mitotic process (Table 2). Indeed, although dietary-modulated transcripts in ATs were not primarily enriched in lipid metabolism, individual genes clustered in the response to hormone stimulus had known relationships with lipid metabolic process: the cytosolic enzyme malic enzyme (*ME1*) that generates NADPH for fatty acid biosynthesis and which was down-regulated > 2 fold (BH-*p* value < 0.01) by the HF diet, the leptin (*LEP*) coding for a protein secreted in ATs to regulate food intake and energy expenditure and which exhibited > 1.5-fold reduction in its expression level in HF pigs (BH-*p* value < 0.001), the low-density lipoprotein receptor (*LDLR*) reduced by > 1.4-fold (BH-*p* < 0.001) by HF diet, and the adiponectin receptor (*ADIPOR2*) that mediates lipid oxidation and was also significantly lowered (BH-corrected *p* < 0.001) for pigs fed the HF diet. Conversely, *IGF1R* coding for the insulin-growth factor 1 receptor binding IGF-I, a well-known regulator of cell development, and which was first listed in the significantly-enriched phosphorus metabolic process, was also included in the less enriched clusters corresponding to the response to hormone stimulus and the regulation of cell death; this gene was one of the top genes being up-regulated by HF diet. Altogether, different genes were thus listed in more than one of these pathways, suggesting that these various biological processes were at least in part, inter-connected.

### Particularities of perirenal adipose tissue response to diet

To provide another representation of the transcriptional changes in ATs response to diet, the Weighed Gene Correlation Network Analysis (WGCNA) was used to capture strong relationships between transcripts in modules of interconnected genes [23]. In each module, the eigengene (the weighed mean of the transcripts providing the best univariate summary of the within-module variability) was calculated to relate transcriptional changes to external phenotypic traits and to deduce the biological meaning of the module. Four distinct network modules were thus obtained (Table 3).

**Table 3:** Co-expressed gene networks in adipose tissues in response to diet

Inter-dependent modules	Number of DEP in each module		
	Total	Of PRAT	Of SCAT
Turquoise^a^	6,720	5,302	1,418
Blue	2,738	1,416	1,322
Brown	1,238	79	1,159
Yellow	973	931	42

^a^Five co-expressed gene network modules were deduced from the dataset of differentially-expressed probes (DEP) in response to diet (high-fat high-fiber *vs.* low-fat high starch) and obtained in perirenal (PRAT) and subcutaneous (SCAT) adipose tissues. The total number of DEP included within each module and the number of DEP from each tissue in the module were indicated

The first two big modules represented 48 % and 20 % of the DEP, respectively. The first module (turquoise) included a higher number of DEP from PRAT than from SCAT, and the second one (blue) had almost an equal number of DEP from the two adipose tissues. Eigengenes in the turquoise and blue modules were highly correlated to the first comprised dimension Dim1 in MFA (data not shown), suggesting that these modules included not much more supplementary biological meaning than that deduced from Dim1. Two smaller network modules represented 11 % and 7 % of the DEP data set, respectively. The brown module corresponded mainly to a co-expression network in DEP from SCAT; however, most of these DEP were also found in PRAT including gene transcripts related to protein catabolic process, protein transport, pyruvate metabolism and cell respiration. The pattern of expression in the yellow module was mainly assigned to PRAT; only 4 % of the DEP in this module were also listed as differentially-expressed in SCAT in response to diet. In addition, the eigengene of this yellow module was not highly correlated with %PRAT (r = −0.25; p = 0.09), suggesting that molecular mechanisms unrelated to adipose expansion were predominant in this module. Thus, biological particularities at the PRAT location in the dietary-associated responses can be revealed by further examining the interconnected genes in the yellow module.

A total of 973 DEP corresponding to 411 unique DEG were listed in the yellow module. Most of the DEP (969 out of 973) were up-regulated by the HF diet (see Additional file 3: Table S3). Altogether, 207 DEP corresponding to 91 unique DEG, all up-regulated by the HF diet, were highly correlated to the eigengene of the yellow module (r > |0.70|, *p* < 0.001), so that they could be considered as very relevant for the biological meaning of this module. Functional clustering of these 91 DEG revealed defense response including inflammatory and innate immune responses, membrane organization, natural killer cell mediated cytotoxicity, and protein amino-acid phosphorylation as the top-enriched biological processes (Table 4); the detailed list of genes within each cluster can be found in Additional file 4: Table S4.

**Table 4:** Biological meaning of co-expressed genes in perirenal adipose tissue of pigs

Biological Process	Nb of genes	E score	*p*-value	HF vs. LF diet
GO:0006952 ~ defense response	21	6.96	<0.001	↑
(GO:0006954 ~ inflammatory response)	(15)			
(GO:0045087 ~ innate immune response)	(10)			
GO:0016044 ~ membrane organization	11	3.92	<0.001	
(GO:0006897 ~ endocytosis)	(10)			
GO:0045087 ~ innate immune response	10	3.27	<0.001	
(GO:0006956 ~ complement activation)	(5)			
hsa04650:Natural killer cell mediated cytotoxicity	9	2.54	<0.001	
(hsa04664:Fc epsilon RI signaling pathway)	(6)			
GO:0006468 ~ protein amino acid phosphorylation	11	2.34	0.004	
(GO:0007243 ~ protein kinase cascade)				

^a^Unique annotated genes being highly correlated (correlations > |0.70|) to the eigengene of the yellow small module (Table 3) were inferred for their gene ontology (GO) terms for biological processes. Functional clusters having enrichment (E) score > 2 and modified Fisher exact *p*-value less than 0.01 were indicated. All genes were up-regulated (↑) in perirenal adipose tissue when pigs were fed a high-fat high-fiber (HF) diet compared with a low-fat high-starch (LF) diet

Intra-modular connectivity can measure how connected a gene is to the other genes of its module. Intra-modular connectivity values pointed the following genes as among the 20 first most important nodes in the yellow module (Table 5): the cytokine interleukin-10 *(IL10*) and its receptor (*IL10RA*) which are involved in anti-inflammatory response, three q sub-components of the complement component 1 system (*C1QA, C1QB, C1QC*) and the cytochrome b-245 (*CYBB, CYBA*) which are involved in reactions participating to innate immunity, arrestin beta-2 (*ARRB2*) causing specific dampening of cellular responses to stimuli, and a member of the VAV gene family (*VAV1*) as a natural killer playing role in T-cell and B-cell development.

**Table 5:** Most important genes participating to particularities in response to diet of perirenal adipose tissue

Gene symbol	Full name	Connectivity^a^
*IL10*	Interleukin 10	205.8
*C1QB*	Complement component 1, q subcomponent, B chain	204.9
*CYBB*	Cytochrome b-245, beta polypeptide	201.9
*C1QC*	Complement component 1, q subcomponent, C chain	201.9
*C1QA*	Complement component 1, q subcomponent, A chain	199.2
*ARRB2*	Arrestin, beta 2	195.2
*CYTH4*	Cytohesin 4	194.8
*CYBA*	Cytochrome b-245, alpha polypeptide	194.7
*NCKAP1L*	NCK-associated protein 1-like	193.9
*FCER1G*	Fc fragment of IgE, high affinity I, receptor for gamma polypeptide	193.3
*VAV1*	Vav 1 guanine nucleotide exchange factor	192.9
*FOLR1*	Folate receptor 1	191.6
*CD53*	CD53 molecule	190.9
*VSIG4*	V-set and immunoglobulin domain containing 4	190.6
*IL10RA*	Interleukin 10 receptor, alpha	189.2
*CFP*	Complement factor properdin	188.8
*VSIG4*	V-set and immunoglobulin domain containing 4	188.2
*CADM1*	Cell adhesion molecule 1	188.1
*NCF4*	Neutrophil cytosolic factor 4, 40 kDa	187.8
*F13A1*	Coagulation factor XIII, A1 polypeptide	187.7

^a^The first 20 genes were ranked according to their connectivity value within the yellow module, which assembled a total of 411 unique genes in perirenal adipose tissue, all up-regulated by a high-fat high-fiber diet. The higher is the connectivity value, the stronger is the evidence that the gene is part of the module

### Upstream regulatory candidates

The two above-described analyses have clearly shown that different biological pathways were involved in responses to diet; therefore, it is important to identify upstream regulators which coordinate these molecular changes in porcine ATs. For this purpose, the transcripts of genes identified as differentially-expressed by diet within each AT were submitted to a dedicated algorithm (http://keyregulatorfinder.genouest.org/) which matches the lists of DEP to a causality graph of reactions and regulations built from Transpath® database information, and ranks upstream candidates in accordance to their ability to regulate significant numbers of the DEP. The obtained candidates were listed in Additional file 5: Table S5 for PRAT and in Additional file 6: Table S6 for SCAT. A biological post-prioritization in these candidates was manually made by adding GOBP terms. Only a summary of the most relevant candidates was given in Table 6, but more background information on the biological processes in which the candidates may play a role is provided in Additional file 5: TableS5 and Additional file 6: Table S6.

**Table 6:** Candidates for upstream regulatory roles in functional pathways altered by diets

Genes proposed as regulatory candidates^a^	Associated biological processes
*SREBF1, MLXIPL, PPARδ, PPARα, PPARγ, USF1, OGT, GALNT1, PPP1R1A, PPP1R3C, B4GALT5, FGFR4*	Glucose metabolic process
*SREBF1, MLXIPL, PPARδ, PPARα, PPARγ, RXRA, RXRG, SREBF2, GATA4*	Co-factor metabolic processes related to lipids and cholesterol
*RXRA, RXRG, RARA, OGT*	Response to organic substances
*MLXIPL, GLOD4, NRF1, PPARGC1A, ACOX2*	Phosphorus metabolic process
*PPARδ, OGT, NFE2L2, RARA, MEF2A, MEF2C, MEF2D, ASNS, ZNF268, EBAG9, DLC1, MASP1*	Regulation of apoptosis and cell death
*PPARδ, MLXIPL, MAFG, KLF13, CCDC85B, ZNF268, IKZF1, COPS2, NFYA*	Cell cycle process
*OGT, GALNT1, NFE2L2, UBL4A, ASNS, NR1H3, B4GALT5, RFN10, ZNF268, FEM1A, TOMM7*	Protein metabolic process
*SREBF1, UBL4A, TOMM7*	Protein transport
*PPARδ, NRF1, NFE2L2, PPARGC1A*	Response to stress
*PPARα, PPARγ, NFE2L1, IL25*	Defense response, inflammatory response
*PPARγ, MEF2A, CNIH, MASP1, FGFR4*	Innate immune response

^a^The lists of differentially-expressed genes in porcine perirenal (PRAT) or subcutaneous (SCAT) adipose tissues in response to diet were automatically confronted to the academic information on regulations and reactions in mammalian cell signaling. Genes that were able to regulate a significant number of the differentially-expressed genes were proposed as upstream regulatory candidates. The biological processes in which these genes could be involved were indicated as referenced in the gene ontology (GO) Entrez database. The complete lists of candidates are given in Additional file 5: Table S5 for PRAT and Additional file 6: Table S6 for SCAT

**Fig. 3:**
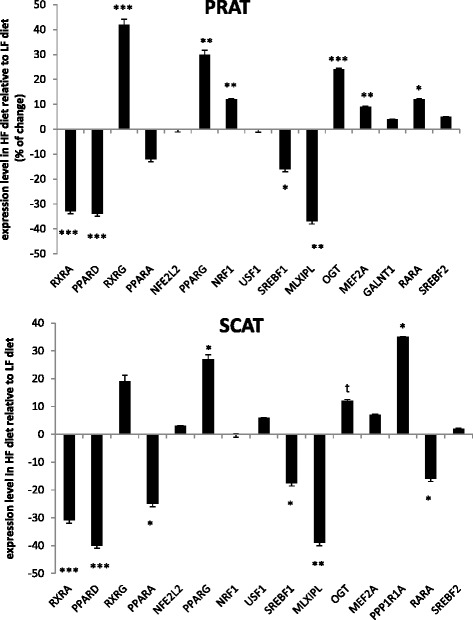
Expression levels of transcription factors examined by qPCR as affected by diet in adipose tissues. The %variation ratio in expression level of each target gene was shown for HF diet (high-fat high-fiber) relative to LF diet (low-fat high-starch) for perirenal (PRAT) and subcutaneous (SCAT) adipose tissues. When a gene was down-regulated by the HF diet, the value was preceded by a minus sign. ***p < 0.001; **p < 0.01, *p < 0.05, ^t^0.05 < *p* < 0.10

Dietary-related changes in expression levels of 16 genes were studied by qPCR in the 48 samples of PRAT and the 48 samples of SCAT to provide a functional validation among the graph driven-hypothesis upstream candidates. In the two ATs, the majority of the examined candidates were significantly affected by diet (Fig. 3).

This included the under-expression by the HF diet of the MLX interacting protein-like *MLXIPL* (alias *ChREBP*) and the sterol-regulatory binding protein-1 (*SREBF1*), two transcriptional factors known to regulate glucose and lipid metabolism. The peroxisome proliferator activated receptor delta (*PPAR*)*δ* and the retinoic X receptor alpha *RXRA* forming heterodimers to bind promoters of target genes in energy metabolism and apoptotic process were also down-regulated by the HF diet in the two ATs; however, *PPARα* isotype was confirmed as under-expressed (*p* < 0.05) only in SCAT. *PPARγ*, the last isotype of the PPAR family having pleiotropic functions in lipid metabolism and adipocyte differentiation was conversely up-regulated by the HF diet in the two ATs. It is noteworthy that the direction of change in *PPARγ* mRNA levels between HF and LF diet by qPCR was inconsistent with the sign of variation predicted by the algorithm (Additional file 5: Table S5), so that *PPARγ* was regulated by diet in a way opposite to the two other PPAR isotypes. The O-linked N-acetylglucosamine transferase *OGT* regulating protein ubiquitination and cell death and the protein phosphatase 1 regulatory subunit 1A (*PPP1R1A*) known to regulate glycogen metabolism were also confirmed as being up-regulated by the HF diet. Surprisingly, the retinoic acid receptor alpha (*RARA*) involved in apoptotic process was regulated in an opposite manner between the two ATs. Specificities for PRAT were further enlightened for three upstream candidates that were over-expressed in HF pigs, without any significant variations in their expression levels compared with LF pigs in SCAT: *RXRG* involved in mediating the anti-proliferative effects of retinoic acid, the nuclear respiratory factor *NRF1* involved in cell response to stress, and the myocyte enhancer factor *MEF2A* involved in innate immune response. Finally, the relative expression levels of *USF1, SREBF2, NEF2L2* and *GALNT1* did not differ between diets in the two porcine ATs. Except for *GALNT1* in PRAT and *NFE2L2* in SCAT, this was in agreement with microarray data (Additional file 5: Table S5 and Additional file 6: Table S6) which indicated no significant variations in these gene transcripts when pigs were fed HF or LF diets. We observed that sirtuin 1 (*SIRT1*) was not proposed as an upstream regulatory candidate of the AT transcriptomes, although this gene currently attract much attention in the literature; a detailed examination of the list of DEG revealed its up-regulation by the HF diet notably in PRAT (x1.25 fold as compared with LF diet, BH-adjusted *p* < 0.03).

## Discussion

Irrespective of species, there are still a limited number of studies which have deciphered the effects of changing dietary energy source rather than dietary energy level on gene expression levels in AT [7, 24, 25]. A recent study on Iberian pigs revealed only minor influences of dietary energy source (sunflower oil and fiber opposed to carbohydrates) on dorsal subcutaneous fat transcriptome [26]. In the present study, feeding pigs a diet rich in lipids (rapeseed and soybean oils) and fibers (insoluble wheat-straw) during 58 days led to 1.4-fold reduction in adiposity as opposed to pigs fed a ration characterized by starch as the main energy source. Large numbers of genes in perirenal AT and, to a lesser extent in subcutaneous AT, exhibited dietary-related changes in expression levels. Complementary integrative statistical methods and functional bioinformatics tools were thus used on the transcript data sets to identify the most important metabolic pathways accounting for dietary-related variations adiposity across the two ATs.

### Depressed catabolic protein process and lower glucose use for anabolic pathways were primarily associated with reduced body adiposity by a high-fat high-fiber diet

Among the 436 unique genes which exhibited close-similar dietary-induced variations in their expression levels across the two ATs, protein catabolic process was suggested as the top- down-regulated pathway acting in adiposity variation by diet. Down-regulation of the ubiquitin-mediated proteolysis and proteasome pathway has been previously reported in the liver for a specific strain of mice fed a high-fat diet compared with a carbohydrate chow diet [27]. In the present study, it is possible that molecular changes related to protein catabolism have also resulted from the introduction of insoluble fiber in the pig diet, which may have compromised dietary protein digestibility and nitrogen retention [28]. The finding of lower plasma level of lysine, an indispensable amino-acid, in pigs fed the HF diet as compared with pigs fed the LF diet in our associated study [29] supports this hypothesis. Although the relationships between potential changes in the ubiquitin-proteasome system and AT development cannot be fully elucidated, roles in the regulation of proteins involved in cell division, cell death and signal transduction [30] could be inferred. Indeed, dietary-related modulations in groups of genes regulating apoptosis and cell cycle progression were observed across ATs in the present study. Altogether, finding reduced expression of genes coding for proteasome subunits in ATs of the leanest HF pigs is consistent with data in murine 3 T3-L1 adipocytes showing that proteasome inhibition led to impaired adipocyte differentiation and decreased lipid content [31].

Considering the differences in lipid and starch contents between HF and LF diets, the modulation of several gene transcripts related to glucose metabolism in the two ATs was expected. Different glucose anabolic pathways were suggested to be repressed by the HF diet, likely due to the down-regulation of hexokinase 2 (*HK2*) which catalyzes the first step in glucose metabolic pathways [32]. Even though AT contain only traces of glycogen, finding depressed transcriptional levels of different genes related to glycogen synthesis in HF pigs suggests that variations in glycogen metabolic enzymes may be produced by nutritional treatments in porcine AT as previously observed in the rat [33]. It is reasonable to speculate relationships between glycogen metabolism in AT and adiposity variation, considering recent findings showing that knocking-down glycogen synthase kinase 3 beta (*GSK3B*) inhibits preadipocyte differentiation [34]. The lower expression levels of the pyruvate kinase and different members of the pyruvate dehydrogenase complex, which were observed in the two ATs of HF pigs, suggest that the outflow from the glycolytic pathway to mitochondria might be also reduced in these pigs. At the molecular level, these observations fit with the depressed basal activity of pyruvate dehydrogenase reported in AT of rats adapted to a high fat diet compared with rats fed a high glucose diet [35]. In contrast with human and rats, the porcine AT is considered the main lipogenic site [36], so that glucose utilization and *de novo* lipid synthesis are inextricably intertwined in pig ATs. Therefore, the down-regulations of genes participating to co-factor metabolic processes such as ATP-citrate lyase (*ACLY*) responsible of cytosolic acetyl-CoA production and malic enzyme (*ME1*), the rate-limiting enzyme in *de novo* lipogenesis in the pig [37], suggest that lipid synthesis from carbohydrates was reduced in ATs when feeding pigs the HF diet. Less obvious, the down-regulation of 3-hydroxy-3-methylglutaryl-CoA reductase (*HMGCR*) may reduce cholesterol synthesis in ATs of HF-fed pigs. In agreement, interactive effects between dietary fat and dietary fiber have been reported with regard to cholesterol-related traits in pig AT [38].

### No reciprocal stimulation of lipid metabolism occurred in ATs when lipids and insoluble fibers were included in the diet

Usually, inhibiting lipogenesis does not disrupt AT growth if extracellular lipids are available. In the present study, we did not find any specific enrichment in lipid-related pathways among genes regulated by diet. However, the low-density lipoprotein receptor (*LDLR*), which can directly internalize triglyceride-rich lipoprotein remnants as whole-particles into adipose cells [39], was down-regulated by the HF diet. Due to the wheat-straw-derived fiber consumption, part of the dietary lipids may have been sequestrated into the viscera and subsequently reduced quantities are readily to provide energy to sustain fat development in HF pigs. An increase in the weight of the digestive tract and increased hepatic lipid content in HF pigs compared with LF pigs support to this hypothesis [21]. Furthermore, the *LEP* gene encoding leptin, an adipokine secreted by ATs to regulate food intake and energy expenditure, was found down-regulated in ATs of HF pigs, an observation which was in agreement with the reduced circulating concentration of leptin reported in our associated study on the same pigs [29]. Finally, due to the down-regulation of its receptor (*ADIPOR2*) in ATs of HF-fed pigs, it is tempting to speculate that there is an alteration to adiponectin signaling, another adipocyte-related protein that may facilitate adipose expansion in situation of extra-nutrients supply [40]. Direct measurements of circulating adiponectin concentrations and of *ADIPOR1/2R* in skeletal muscle and liver are however needed to unravel the significance of this change with regard to the control of carcass fat and insulin signaling.

### Upstream transcriptional factors were suggested to act on metabolic flexibility in ATs in response to contrasted dietary nutrients

As each gene can participate in more than one biological pathway and because many biological processes are highly inter-connected, it is of interest to decipher the main upstream regulators in the molecular pathways participating in the observed limitation of fat expansion by the HF diet. The influence graph-driven qPCR validation provides evidence for the carbohydrate responsive element binding protein ChREBP also known as MLX-interacting protein-like (*MLXIPL*), as a pivotal transcriptional mediator down-regulated by the HF diet in porcine ATs. This was expected considering that this gene is conversely activated in response to high glucose (like in LF diet) to up-regulate several genes involved in the metabolic conversion of glucose to fat in the rodent liver [38]. Moreover, glucose-related stimulation of lipogenic gene expressions through ChREBP has been recently demonstrated in porcine primary adipocytes [41]. Another classically-recognized gene regulator controlling the expression of nearly all genes integral to glucose use for anabolic purpose [42, 43] is the sterol-regulatory element binding protein-1 (*SREBF1*), which was lesser expressed in ATs when pig were fed the HF diet. Down-expression in *SREBF1* expression by an increased dietary fat intake irrespective of the type of fibers included in diet was similarly revealed in porcine subcutaneous AT [7]. This gene is responsive to increased insulin levels associated with consumption of high carbohydrate diets, so that its reduced expression in ATs was in agreement with the reduced insulinemia in the HF pigs reported in our associated study [29]. Conversely, finding an over-expression of O-GlcNAc transferase (*OGT*) in the two ATs of HF pigs is worthy of interest. Indeed, this gene encodes the enzyme responsible for adding O-linked-β-*N*-acetyl-glucosamine to proteins, thus affecting ubiquitination and subsequent protein degradation. Increased *OGT* mRNA expression has been reported in response to glucose starvation in mammals [44], a nutritional situation that is partly comparable to feeding a HF diet. Finally, differential regulation of PPARs binding fatty acids and their derivatives were suggested in the present study. PPARs are nuclear hormone receptors expressed in three isoforms (α, γ, δ) and each PPAR forms a heterodimer with RXR. While *PPARα* is highly expressed in tissues with a high rate of fatty acid oxidation, *PPARγ* is considered as an adipocyte predominant transcription factor and *PPARδ* is ubiquitously expressed. In the two pig ATs, the variations observed in HF vs. LF diet were opposite to those reported in mice AT where the consumption of fibers induced rather than decreased *PPARδ* expression [24] and decreased rather than increased *PPARγ*-activated processes [45, 46]. In support to the present results, short-chain fatty acids such as butyrate however enhance *PPARγ* expression in the stroma vascular fraction isolated from porcine AT [47]. Similarly to our results, pigs fed a diet enriched in oil and fibers also exhibited up-regulation in *RXRG*, a molecule narrowly related to *PPARγ* expression, when compared with pigs fed carbohydrate-based diet [26]. Because of their pleiotropic functions in the regulation of glucose and lipid metabolism, oxidative stress and inflammation, the reasons why *PPARδ* and *PPARγ* may be oppositely regulated by the diet in porcine AT deserve further studies.

### Adiposity-unrelated modulation in inflammation and innate immune responses by a high-fat high-fiber diet was underlined in the perirenal fat

The composition of fatty acids (unsaturated or saturated) in ATs of pigs generally reflects the dietary fatty acid pattern when fed a high-fat diet and *de novo* lipogenic intensity when fed a low-fat high-starch diet [21, 26]. The nature of fatty acids together with other food compounds may contribute to immune activation and chronic inflammation, irrespective of adiposity variations. In the present study, a module of interconnected dietary-regulated genes that were poorly related to adiposity mass variation but with clear links to defense mechanisms, including immunity and inflammation, was strengthened in PRAT. Among these genes, *CYBA/CYBB* genes coding for chains of the oxidase system, a significant contributor to the pathogenic effects of high-fat diets [48], were up-regulated by the HF diet. Conversely, many up-regulated transcripts in PRAT of HF pigs encoded proteins with anti-inflammatory properties, such as interleukin-10 (*IL10*) and its receptor *IL10RA*. This observation is in agreement with another study in human AT showing of *IL10* activation after ingestion of purified dietary fibers [49]. The mRNA levels of *C1QA/B/C* genes were also higher in HF pigs. Because the complement C1q is generally protective [50], this may be an argument to support a positive modification in the outcome of inflammation by the HF diet. Similarly, *VAV1*, a key negative regulator of macrophage-derived pro-inflammatory cytokine production [51], was up-regulated in PRAT of HF pigs, suggesting that this benefit may have concerned infiltrated macrophages rather than adipocytes. Altogether, these observations suggest that dietary fat and fiber had antagonistic actions on inflammatory and anti-inflammatory pathways in PRAT. In support, fiber-rich food consumption attenuated the inflammation induced by the intake of a high-fat diet in the liver [52]. In AT, the downregulation of genes related to inflammation and immune response has been reported in response to the intake of a diet rich in fat and viscous non-fermentable soluble dietary fiber [25]. The modulations in inflammation and immune pathways were the most striking example of contrasting transcriptional adaptation to diets between PRAT and SCAT, as the above-mentioned genes were unchanged in SCAT. In agreement with this finding, porcine visceral AT has previously been shown to be enriched in miRNAs associated with immune and inflammation responses when compared with subcutaneous AT [20]. Of interest in this study, the nuclear respiratory factor 1 (*NRF1*) was up-regulated by the HF diet in PRAT only. While this target is involved in a wide range of processes including proliferation, oxidative stress, apoptosis and innate immune response, long-term *NRF1* overexpression in 3 T3-L1 adipose cells stimulated cytokine expression such as clusters of differentiation attracting macrophages [53]. The graph-driven identification of upstream regulatory candidates did not propose *SIRT1*, a master transcriptional regulator of both metabolism and inflammation in response to states of energetic excess and high-fat feeding [54]. Because *SIRT1* was found up-regulated in PRAT by HF diet, its role in the protection against pro-inflammatory molecular responses might have been confounded by processes related to PPARs [55]. Moreover, not all changes take place at a transcriptional level, so that functional studies are needed to clarify the role of SIRT1 protein in porcine AT. Altogether, the side effects of the HF diet on the modulation of the inflammasome in PRAT may be important to consider in conditions where chronic inflammation plays an important mitigating role and is contrary to optimal health and growth performance.

## Conclusion

This study presents novel findings on the molecular modulations related to dietary energy source and nutrients in two adipose tissues of growing pigs. Particularities in the dietary responses for immunity and inflammation features are again enlightened at the perivisceral location.

## Methods

### Ethic statement

The care and use of pigs were performed following the guidelines edited by the French Ministries of High Education and Research, and of Agriculture and Fisheries (http://ethique.ipbs.fr/sdv/charteexpeanimale.pdf). The protocol was approved by the local Ethics Committee in Animal Experiment of Rennes, France (Comité Rennais d’Ethique en matière d’Expérimentation Animale, CREEA, http://ethique.ipbs.fr/creeapresent.html; agreement N°R-2012-07). All pigs were reared and killed in compliance with national regulations and according to procedures approved by the French Veterinary Services at INRA Pegase experimental facilities.

### Animals and diets

Data from a total of 48 pigs (pure Large White breed) were considered in the present study. These pigs originated from two lines divergently selected over eight generations for residual feed intake (RFI) as a measure of feed efficiency [56]. The RFI index was typically calculated as the difference between actual and predicted feed intakes, so that low RFI value signified better efficiency in the conversion of food to weight gain. In this experiment, barrows originated from the selection herd (INRA GeneSI, Le Magneraud, France) were transferred after weaning (pig age: 28 days) at INRA Pegase (St-Gilles, France). From 74 d ± 0.3 d of age onwards, pigs were randomized by line in two dietary groups (*n = 12* per diet and per line): a high-fat high-fiber diet (HF) or a low-fat high-starch diet (LF). All pigs had free access to its experimental regimen during 58.5 ± 0.5 days. The diets were formulated in two formulas distributed during the growing and finishing periods, respectively, and for which nutritional composition (protein content and energy values) followed recommendations for an optimal growth during these two periods. Importantly, the HF and LF diets included similar crude protein contents (17 % and 13 % for growing and finishing formulas, respectively) and digestible essential amino acids (lysine, methionine and threonine). Starch derived from cereal grains (wheat and barley) in the LF diet was partially replaced by rapeseed and soybean oils in the HF diet and crushed wheat straw (insoluble fiber) was included as a diluent of dietary energy in this diet, so that LF and HF diets can be formulated at an isocaloric basis (12.9 MJ/kg metabolizable energy). This strategy resulted in large variations between HF and LF diets in their nutritional composition (as fed basis): respectively, 33.5 % vs. 45.5 % of starch, 7.2 % vs. 2.2 % of fat, 18 % vs. 12.1 % of neutral-detergent fiber and 8.2 % vs. 3.6 % of acid-detergent fiber. Full composition of diets has been detailed elsewhere [21].

At 132.5 d ± 0.5 d of age, pigs were euthanized by electric stunning and exsanguinated. Samples were immediately collected at the dorsal subcutaneous adipose tissue (SCAT) location (last rib level) by an incision made along the dorsal right side of the body. After opening the abdominal cavity by a ventral incision, the perirenal adipose tissue (PRAT) was totally collected and weighed. It was immediately cut in small pieces, which were frozen in liquid nitrogen and stored at −70 °C until analysis. The day after, the dorsal SCAT was entirely removed from the left side of the carcass and weighed. Weights of PRAT and SCAT were expressed as relative to pig live weight (%PRAT and %SCAT, respectively).

### RNA isolation and reverse transcription

Total RNA was extracted from ATs (80 to 100 mg each) by homogenization in Trizol reagent (Invitrogen, California, USA) and then, purified using a silica-membrane technology under vacuum (Nucleospin® 8 RNA kit, Macherey-Nagel, France). The method included a DNase digestion step to remove contaminating DNA. The total RNA was eluted in 50 μl of RNase-free water and stored at −70 °C. The quantification of RNA was performed by using a NanoDrop® ND-1000 spectrophotometer (Thermo Scientific, Illkirch, France). Ratios of A260/280 and A260/230 were greater than 1.7 in all samples, denoting good purity. The RNA quality was verified using the Agilent RNA 6000 Nano kit and an Agilent 2100 Bioanalyzer (Agilent Technologies France, Massy, France). Average RNA integrity number was 8.3 ± 0.5.

### RNA labelling and microarray hybridization

The custom porcine microarray (8 × 60 K, GPL16524, Agilent Technologies France, Massy, France) used contain 60,306 porcine probes derived from the Agilent commercial 44 K microarray (V2:026440, GPL16571) and enriched with immune system, muscle and adipose tissue genes. About 57,387 probes are annotated. It is important to consider that annotations are being constantly improved and that annotations of all cited genes were carefully checked.

Each sample was labeled with Cy3 dye using the Low Input Quick Amp Labeling kit (Agilent Technologies) following the manufacturer’s instructions. Briefly, a two-step procedure generates fluorescent complementary RNA (cRNA) by using T7 RNA polymerase, which simultaneously amplified target and incorporated cyanine-labeled CTP. Samples were then purified with an RNeasy Mini kit (Qiagen, Hilden, Germany). Microarray hybridizations were carried out in Agilent’s hybridization chambers containing 600 ng of Cy3-labeled cRNA sample per hybridization. Hybridization reactions were performed at 65 °C for 17 h using Agilent’s Gene Expression Hybridization Kit. After washing, microarrays were scanned at 3 μm/pixel resolution using the Agilent DNA Microarray Scanner, and images were analyzed with Agilent Feature Extraction Software (Version 10.7.3.1, protocol GE1_107_Sep09).

Microarray analyses were performed using the R software version 2.10.0 [57]. Raw spots intensities were first submitted to quality filtration based on 4 criteria: intensity, uniformity, saturation and outliers detection. Intensities of filtered spots were transformed into log2(Cy3), and data were normalized by median centering, i.e. subtracting the median value across all probes from all raw values for each sample in order to obtain experimentally consolidated gene expression values. Microarray selected data have been deposited in NCBI’s Gene Expression Omnibus (GEO) and are accessible through GEO subseries accession number GSE70836 (http://www.ncbi.nlm.nih.gov/geo/query/acc.cgi?acc=GSE70836) for SCAT and GSE70837 (http://www.ncbi.nlm.nih.gov/geo/query/acc.cgi?acc=GSE70837) for PRAT.

### Statistical analyses

In each AT, the data were first analyzed by a two-way analysis of variance (ANOVA) with diet (HF *vs.* LF), line (low *vs.* high RFI) and the interaction between diet and line as the main effects. A raw *p*-value < 0.01 and fold-change between conditions > |1.1| were considered as cut-offs [58] in a heuristic way to retain differentially-expressed probes (DEP). Data were also submitted to the Benjamini-Hochberg (BH) multiplicity correction of the *p*-values for a control of the FDR at level 0.08 [59], proving stringent control of statistical errors. Within each AT, the lists of DEP, one for diet and one for line, were thus edited. Venn diagrams representing the number of DEP in the two ATs as affected by diet or by line were produced.

Second, to derive a common framework that might be more convenient to highlight communalities across the two ATs, the Multiple Factor Analysis (MFA) was used as a data integration statistical method [12] under the FactoMiner package in R software. Briefly, two principal component analyses (PCA) were first performed from the DEP dataset obtained in each adipose tissue (one for PRAT, one for SCAT). Each variable identified by the probe name was weighed by the first eigenvalue in PCA. Then, the analysis consisted in realizing a meta-PCA by using these weighed variables as new entries, so that each tissue influence was equally represented. The first two MFA components (Dim1; Dim2) were linked to the three first axes of the two separate PCA (Dim_i_PRAT and Dim_i_SCAT with *i* = 1 to 3) by calculating the correlations between MFA and PCA components. This allows building a MFA diagnostic plot to study the relationship between the observations, the variables and the tables, and which facilitates the description of communalities. Adiposity phenotypic traits (%_SCAT and %_PRAT, respectively) were superimposed on the plot to relate variations in transcriptomic profiles to changes in body fat. Calculating the correlations between each DEP and MFA dimensions (Dim_1; Dim_2) allows deciphering the DEP that were mainly responsible for communalities in molecular responses between the two ATs; the threshold r > |0.70| (*p* < 0.001) was retained to list these main contributors.

Third, global gene co-expression networks were constructed from the dataset joining the DEP of the two ATs, with the main goal to find modules of genes with similar transcriptional profiles [23]. The WGCNA package in R software, which has been described as one of the methods that performed the best for describing the correlation patterns among genes, was used. As recommended [60], a power (s, β = 6) was retained to strengthen the separation between low and high correlation in gene expression levels within the modules. Clustering methods were implemented on the adjacency matrix (encoding the connection strength between pairs of nodes) to extract network modules of co-expressed genes. A single virtual gene expression termed the eigengene can be calculated to summarize the gene expression profile within the cluster. An equivalent definition can be given where the module eigengene is defined as the first principal component in PCA [23]. To have a measure of how closely related a particular actual gene is to the eigengenes, a correlation coefficient was calculated between each individual gene in the module and the eigengene. In the present experiment, only DEP highly correlated (r > |0.70|; *p* < 0.001) to the eigengene in each module were further considered to deduce its biological meaning. Intra-modular connectivity was used as an additional network concept; the higher is the connectivity value, the stronger is the evidence that the gene is a central node of the module. Finally, eigengenes of the different clusters were used to explore the associations between each module and MFA dimensions, and between each module and adiposity phenotypic traits (%PRAT, %SCAT).

### Functional pathways analysis

The most important DEP (r > |0.70|) participating in the comprised MFA dimensions or in the WGCNA modules were considered for functional analyses. The correspondence between DEP and its official gene symbol was first made, when applicable. The gene ontology (GO) terms for biological processes were then automatically searched, using the Database for Annotation, Visualization and Integrated Discovery (DAVID) bioinformatics resource database (http://david.abcc.ncifcrf.gov/). The GOBP terms_FAT were selected to filter the broadest terms without overshadowing the more specific ones. The results were downloaded using the “Functional annotation clustering” option of the DAVID tool. The enrichment score (measured by the geometric mean of the EASE score of all enriched annotations terms) and the modified Fisher exact *p*-value, were obtained. As recommended [61], an enrichment score E ≥ 1.3 and *p* < 0.05 were first considered to list the significantly top-enriched clusters of genes. However, groups with a lower E score were also indicated as individual genes could be also potentially interesting.

### Upstream regulatory candidates

A web tool (KeyRegulatorFinder.genouest.org/) that has been recently developed [62], was used to propose upstream transcriptional regulators that may have participated to the molecular flexibility in response to diet within each AT. This methodology aimed to provide a reasonable number of upstream regulatory candidates for the transcriptomic changes, by an automatic confrontation of the DEP dataset with the academic information on regulations and reactions in mammalian signal transduction included in Transpath® database. A causality graph was built across the different molecules, so that genes and(or) gene complexes being able to regulate a significant number of DEP from the input list could be considered as candidates for an upstream regulatory role. The algorithm can also predict whether the upstream candidates were activated or inhibited by the conditions under study. In the present experiment, the DEP to be included in the input list were first identified by their Official Gene Symbol and the sign of variation in their expression levels between the two diets (arbitrary set to “+” when up-regulated in HF vs. LF diets, and to “-” when up-regulated in LF vs. HF diet). The proposed candidates were ranked according to a score of specificity [62], and a biological post-prioritization among the first 50 top candidates was further made by manually adding their functional GO terms.

### Quantitative real-time PCR (qPCR)

Finally, a graph-driven hypothesis qPCR validation was performed on a subset of the upstream regulatory candidates in PRAT and in SCAT of the 48 pigs. For qPCR, first-strand cDNA synthesis was performed with 1 μg of total RNA previously used in microarray analysis, using High Capacity RNA to cDNA Kit (Applied Biosystems, Foster City, USA). Primers were designed from porcine sequences available in Ensembl or NCBI databases using Primer Express® v3.0 software (Applied Biosystems). Detailed information for each primer pair including gene name, gene symbol, primer sequences (forward and reverse) and amplicon size are provided in Additional file 7: Table S7. A StepOnePlus™ real-time PCR system (Applied Biosystems) was used for qPCR. Amplification reactions and disassociation curves were realized as described previously [63]. The TOP2B gene was the most stable reference gene stated by GenNorm algorithm among 3 other tested reference genes, and was used for normalization. For each sample, the normalized expression level was calculated using the formula E_target_^-ΔCq^^(sample - calibrator)^ / E_TOP2B_^-ΔCq^^(sample - calibrator)^ where E is the efficiency calculated from the slope of calibration curve, Cq is the quantification cycle and the calibrator is a pool of all samples. Then, ANOVA was used to determine significant effects of diet, line and the interaction between diet and line, using the R software. Both fold-change in gene expression levels and *p*-value were represented.

### Availability of supporting data

The molecular datasets supporting the conclusions of this article are available in Gene Expression Omnibus repository (GEO, http://www.ncbi.nlm.nih.gov/geo/) through the accession number GSE70836 for subcutaneous adipose tissue (SCAT) and GSE70837 for perirenal adipose tissue (PRAT). Microarray data are MIAME compliant. Other datasets are included within the article and its additional files.

## Additional files

Additional file 1: Table S1. List of differentially expressed probes commonly regulated by diets across perirenal and subcutaneous adipose tissues. (XLSX 207 kb) Additional file 2: Table S2. Summary of the biological processes shared by genes being commonly regulated by diet across adipose tissue. (DOCX 23 kb) Additional file 3: Table S3. List of co-expressed probes within a small module mainly attributed to perirenal adipose tissue related responses to diet. (XLSX 40 kb) Additional file 4: Table S4. Detail list of genes associated with different biological process within a small interconnected module in perirenal adipose tissue of pigs fed a high-fat high-fiber diet. (XLSX 11 kb) Additional file 5: Table S5. Complete list of key upstream regulators proposed in response to diets in perirenal adipose tissue and associated biological processes. (XLSX 18 kb) Additional file 6: Table S6. Complete list of key upstream regulators proposed in response to diets in subcutaneous adipose tissue and associated biological processes. (XLSX 18 kb) Additional file 7: Table S7. Primers used for qPCR analysis. (DOCX 17 kb)

## Abbreviations

AT: adipose tissue
DAVID: database for annotation, visualization and integrated discovery
DEP: differentially expressed probe
DEG: differentially-expressed gene
Dim: dimension
GO: gene ontology
HF: high fat high fiber diet
LF: low fat low fiber diet
MFA: multiple factor analysis
PCA: principal component analysis
qPCR: real-time quantitative polymerase chain reaction
PPAR: peroxisome proliferator activated receptor
PRAT: perirenal adipose tissue
RFI: residual feed intake
RXR: retinoid x receptor
SCAT: subcutaneous adipose tissue
WGCNA: weighed gene co-expressed network analysis
