# Supplementary material for: Molecular alterations induced by a high-fat high-fiber diet in porcine adipose tissues: variations according to the anatomical fat location
Source: BMC Genomics. 2016 Feb 18;17:120. doi: 10.1186/s12864-016-2438-3 (PMC4758018; doi:10.1186/s12864-016-2438-3)
Supplement: Additional file 2: Table S2. — Summary of the biological processes shared by genes being commonly regulated by diet across adipose tissue. (DOCX 23 kb) [file 12864_2016_2438_MOESM2_ESM.docx]

**Additional file S2:** Table S2 Summary of the biological processes shared by genes being commonly regulated by diet across adipose tissue *^a^*

| Over-expressed in HF diet | Under-expressed in HF diet |
| --- | --- |
| **Modification-dependent protein catabolic process** | |
| USP32, CBLB, FBXL5 | UBE2NL, UBE2V2,UBE2J2, UBE2L3, UBE2D2, UBE2W, UBE2G1, UBE3A, UBE2N, UFM1, UFD1L, PSMA1, PSMD12, PSMD1, PSMD7, PSMD8, PSMA5, PSME3, SUMO1P3, SENP5, VCP, CUL4A, RAD23B, FZR1, DERL1, FEM1A, OS9, ZNRF2, MARCH5, KCMF1, TCEB1 |
| **Protein transport** | |
| AP1G2, KLHL2, TLK1, RPGR, ZDHHC17, CBLB, SEC23B, MGEA5 | GSK3B, ATP5D, SNAP29, DERL1, CHMP4B, TIMM17A, TIMM8A, TIMM23B, VPS37A, ARF6, CTSA, CHCHD4, SFT2D1, NUP50, SEC22B, PEX13, SAR1B, STX11, SAR1A, ACTN4, SNAPIN, OPTN, TIMM23, YWHAG, VCP, ARCN1, KPNA3, KPNA2, KPNA1 |
| **Co-enzyme metabolic process** | |
| GSTK1 | ACLY, ME1, DLAT, HMGCR, GLO1, NADK, NAMPT, PANK3, GCLM, MOCS1 |
| **Cell response to stress** | |
| MMS19, HMGB2, DUSP10, TLK1, CCM2, IFI16, FOXN3, JMY, ASTE1, REV3L | UBE2NL, UBE2V2, GSK3B, PPP1R15B, RAD23B, DERL1, SUMO1P3, PTPLAD1, UVRAG, , OS9, REV1, CRYAB, ATF4, CDKN1A, CRKL, VCP, CUL4A, CTSD |
| **Phosphorus metabolic process** | |
| IGF1R, AKAP9, VRK2, VRK3, TLK1, FYN, HGF, TYK2, DYRK1A, NEK7 | HK2, GSK3B, UGP2, ATP5D, PDK1, ATP6V1F, ND1, NADK, PTPLAD1, PI4KAP2, ADRBK2, PXK, UHMK1, UQCR10, CSNK2A1, IRAK2, TWF1, PI4KA, CDK8, MAPK6, LCK |
| **Glucose metabolic process** | |
|  | HK2, GSK3B, GYS2,UGP2, PDK1, PDHA1, PDHA2, DLAT, LEP, MAN2A2, CRYAB, GALM, ATF4 |
| **Protein import** | |
| CBLB | TIMM17A, TIMM23B, TIMM23, PEX13, KPNA1, KPNA2, KPNA3, TIMM8A |
| **Response to hormone stimulus** | |
| IGF1R, HMGB2, MMS19, PRKAR2B, MGEA5, ABCC5 | LDLR, ME1, LEP, ADIPOR2, CRYAB, CDKN1A, RHOQ, BAIAP2, NR0B1, SLC25A36 |
| **Regulation of cell death** | |
| IGF1R, DLC1, SPPL3, IFI16, HGF, JMY, IFT57 | PSME3, PSMG2, VCP, CUL4A, GSK3B, CDKN1A, GLO1, CRYAB, EEF1E1, LCK, CSTB, PRNP TRAF2, TRAF3, TRAF5, DEDD, RRAGA, SOX9, GCLM, PGP, BLOC1S2, PPP2CA, TEX261, API5, ACTN4, CYCS |
| **Mitotic cell cycle** | |
| HGF, RCC1, FOXN3 | PSME3, PSMD7, PSMD8, PSMA1, PSMD12, PSMA5, PSMD1, CUL4A,CDKN1A, FZR1, NOLC1, RRS1, PAFAH1B1, KPNA2, TXNL4A |

^a^A functional analysis was performed to understand the biological meaning behind 436 unique differentially-expressed genes identified as the most important in communalities across adipose tissues in the response to diet. The automatic functional annotation tool DAVID was used to identify biological gene ontology (GO) terms and clustering redundant annotation terms in enriched biological pathways. Genes identified by their official symbol included in the different functional clusters were listed in each pathway.
