# Supplementary material for: Molecular alterations induced by a high-fat high-fiber diet in porcine adipose tissues: variations according to the anatomical fat location
Source: BMC Genomics. 2016 Feb 18;17:120. doi: 10.1186/s12864-016-2438-3 (PMC4758018; doi:10.1186/s12864-016-2438-3)
Supplement: Additional file 7: Table S7. — Primers used for qPCR analysis. (DOCX 17 kb) [file 12864_2016_2438_MOESM7_ESM.docx]

**Table S7** Primers used for qPCR analysis

| Gene symbol | Description | Accession number^1^ | Forward Primer sequence (5’-3’) | Reverse Primer sequence (5’-3’) |
| --- | --- | --- | --- | --- |
| GALNT1 | Polypeptide N-acetylgalactosaminyltransferase 1 | ENSSSCT00000004144 | TGCTCAAATGCCACCACCTA | GGCTTTGTCCAGGCACTGAT |
| MEF2A | Myocyte enhancer factor 2A | ENSSSCT00000028081 | GGAGTTGAATACCCAGAGGATAAGC | AGGCGGTTGGCATTGCT |
| MLXIPL | MLX interacting protein-like | ENSSSCT00000008453 | CGAGGTGGTGATGCGAGAAT | TTGCGGAGCCGCTTCTT |
| NFE2L2 | Nuclear factor (erythroid-derived 2)-like 2 | ENSSSCT00000017408 | GCACAACACATCCCGTCAGA | GAATGTGGGCTACCTGGGAAT |
| NRF1 | Nuclear respiratory factor-1 | XM_005657993.1 | CGGCCTCATGTGTTTGAGTCT | TGGCTCGAAGTTTCCTAAGCA |
| OGT | O-linked N-acetylglucosamine (GlcNAc) transferase | ENSSSCT00000032783 | GTGGGCAAATATTCTGAAGCGT | CTCCTACTGCTGGAAAACGCA |
| PPARα | Peroxisome proliferator-activated receptor alpha | ENSSSCT00000000007 | AAGGTTGCAAGGGCTTCTTTC | CTTACAGCTCCGATCACATTTGTC |
| PPARδ | Peroxisome proliferator-activated receptor delta | ENSSSCT00000001714 | CGCATGAAGCTGGAGTACGA | AGCGAATGGCGTTGTGAGA |
| PPARγ | Peroxisome proliferator activated receptor gamma | ENSSSCT00000012672 | ATTCCCGAGAGCTGATCCAA | TGGAACCCCGAGGCTTTAT |
| PPP1R1A | Predicted: Sus scrofa protein phosphatase 1, regulatory (inhibitor) subunit 1A | XM_005674301.1 | CCACGGCAACAGAAGAAGGT | CCATCATCTGGAGCTCTTTCATT |
| RARA | Retinoic acid receptor alpha | ENSSSCT00000019026 | CAAGACAAATCCTCAGGCTACC | CACCATGTTCTTCTGGATGC |
| RXRA | Retinoid X receptor alpha | DQ279926.1 | GGAGCTGGTGTCCAAGATGAG | CCTTGGAGTCCGGGTTGAA |
| RXRG | Retinoid X receptor gamma | ENSSSCT00000006936 | AATGAAAGACAYGCAGATGGATAAGTC^1^ | TTGGCATCTGGGTTAAATAGCA |
| SREBF2 | Sterol regulatory element binding transcription factor 2 | ENSSSCT00000023268 | GGAGGAGGAGAGCTGTGAATTC | CCCCACAGAGTCCACAAAAGA |
| SREBF1 | Sterol regulatory element binding transcription factor 1 | NM_214157.1 | CGGACGGCTCACAATGC | GCAAGACGGCGGATTTATTC |
| TOP2B | Topoisomerase II beta | ENSSSCT00000012279 | AACTGGATGATGCTAATGATGCT | TGGAAAAACTCCGTATCTGTCTC |
| USF1 | Upstream transcription factor 1 | ENSSSCT00000006981 | TTATTCCCCGAAGTCAGAAGCT | GCGGCGTTCCACTTCATTAT |

^1^ Y=Wobble with mixed bases C or T
